# Diagnostics Using the Change-of-Direction and Acceleration Test (CODAT) of the Biomechanical Patterns Associated with Knee Injury in Female Futsal Players: A Cross-Sectional Analytical Study

**DOI:** 10.3390/diagnostics13050928

**Published:** 2023-03-01

**Authors:** Loreto Ferrández-Laliena, Lucía Vicente-Pina, Rocío Sánchez-Rodríguez, Eva Orantes-González, José Heredia-Jimenez, María Orosia Lucha-López, César Hidalgo-García, José Miguel Tricás-Moreno

**Affiliations:** 1 Unidad de Investigación en Fisioterapia, Spin off Centro Clínico OMT-E Fisioterapia SLP, Universidad de Zaragoza, Domingo Miral s/n, 50009 Zaragoza, Spain; 2 Department of Sports and Computer Science, Faculty of Physical Education and Sports, University of Pablo de Olavide, 41013 Sevilla, Spain; 3 Department of Physical Education and Sports, Faculty of Education, Economy & Technology, University of Granada, 51001 Ceuta, Spain

**Keywords:** knee injury, kinematics, soccer, sports, anterior cruciate ligament, prevention, physiotherapy, CODAT

## Abstract

The primary aim of this study was to identify kinematic differences at initial contact between female futsal players with and without previous knee injury, using a functional motor pattern test. The secondary aim was to determine kinematic differences between the dominant and non-dominant limb in the whole group, using the same test. A cross-sectional study was performed in 16 female futsal players allocated into two groups: eight females with a previous knee injury, i.e., affected by the valgus collapse mechanism without surgical intervention, and eight with no previous injury. The evaluation protocol included the change-of-direction and acceleration test (CODAT). One registration was made for each lower limb, i.e., the dominant (the preferred kicking limb) and non-dominant limb. A 3D motion capture system (Qualisys AB, Göteborg, Sweden) was used to analyze the kinematics. The *Cohen’s d* effect sizes between the groups demonstrated a strong effect size towards more physiological positions in the non-injured group in the following kinematics in the dominant limb: hip adduction (*Cohen’s d* = 0.82), hip internal rotation (*Cohen’s d* = 0.88), and ipsilateral pelvis rotation (*Cohen’s d* = 1.06). The *t*-test for the dominant and non-dominant limb in the whole group showed the following differences in knee valgus: dominant limb (9.02 ± 7.31 degrees) and non-dominant limb (1.27 ± 9.05 degrees) (*p* = 0.049). Conclusions: The players with no previous history of knee injury had a more physiological position for avoiding the valgus collapse mechanism in the hip adduction and internal rotation, and in the pelvis rotation in the dominant limb. All the players showed more knee valgus in the dominant limb, which is the limb at greater risk of injury.

## 1. Introduction

Knee injury incidence is one of the most frequent in soccer, reaching values of 0.7 injuries per 1000 h of exposure [1]. With respect to knee ligament injuries, the anterior cruciate ligament (ACL) is the most common injury, reaching an incidence of 0.45 per 1000 h of exposure, and is also the most common reason for players requiring medical leave of more than 120 days [1,2]. Previous studies have reported that female soccer players have a risk of sustaining ACL injuries that is two or three times higher than the equivalent risk in males [3,4,5], and is usually caused by a non-contact mechanism [6,7], while in men the cause is a direct impact of external force [3,8].

Non-contact mechanisms causing ACL injury are due to a failure in biomechanical patterns, which include a lack of control in proprioceptive and neuromuscular activation [9]. Moreover, it is conditioned by non-modifiable risk factors, such as anatomical [10,11] and hormonal factors [12]. Prospective studies have found that most ACL injuries were caused during dynamic high-intensity stabilization situations such as landings and changes of direction or decelerations, activities that are very common in the defensive roles of pressing/tracking actions in sports [13,14]. In addition, the pressing pattern is one of the most frequent, due to the unexpected stimulus of the opponent and the match context situations [13].

A recent study by DiPaolo et al. [15] showed a compilation of the main biomechanical risk factor thresholds from prospective video analysis studies, which explain the “perfect loading storm” that defines ACL injury mechanisms [6,15,16], valgus collapse being the main biomechanical risk factor related to ACL injury. Arundale et al. [17] explained “valgus collapse” as the combination of hip adduction and internal rotation, and knee abduction. Valgus collapse causes a medial pivoting of the femur on the tibia plateau and compression on the lateral side of the knee. Therefore, an increase in medial yawing of the joint puts the ACL fibres at maximal tension [11,18].

In addition, before the ACL injury appears, on average 48 milliseconds after the initial contact, other kinematics faults have been observed [6,19]. During change-of-direction tasks, at the initial contact phase, the female players who sustained ACL injury showed less trunk, hip, and knee flexion [5,6]. Consequently, this could be related to higher vertical ground reaction forces during impact stabilization. An extended position avoids the load damping of limb joints, which causes an increase in anterior tibial shear forces, potentially resulting in higher strain in the ACL [20]. Zebis et al. [5] indicated a risk reduction of 44% per each increase in 10° of hip flexion at this phase [5]. In addition, studies have considered greater hip and knee rotation movement during change-of-direction tasks as risks of ACL injury [5,21]. Research shows that knee internal rotation at initial contact is significantly related with ACL injury risk, with a 13% increased risk per 1° increase in knee internal rotation. Knee internal rotation was related to higher hip internal rotation during a change-of-direction task, which was considered as a risky movement in terms of non-contact mechanisms causing ACL injury [16,21,22]. Internal hip rotation promotes a displacement of the ground reaction force vector, which is focused medial and posterior on the tibial plateau during the impact. Increased ACL load is caused by the resultant relative anterior and lateral shear of the tibia [16,23,24].

Latest research has studied the incidence ratio based on limb dominance, related to the fact that in futsal the ball determinates the biomechanical pattern [25]. Studies explain that the dominant limb (DL) usually has a higher injury incidence ratio than the non-dominant limb (NDL) [2,25]. During kicking, each limb plays a different role: the DL usually impacts the ball, while the NDL performs a stabilization function to provide a foundation for the kicking task [25]. These different activity profiles influence the load on each limb during kicking, a task that takes place many times during each training session or match [13,26]. This imbalance in loading between the two limbs influences the kinematic response of the limbs to the rest of the tasks in the match, especially in tasks such as changes of direction. Changes of directions have been identified as task related to a high risk of ACL injury.

Most previous studies have analysed the implications of the biomechanical risks factors between two groups, injured and non-injured players [5,27], the former category including injured players who have suffered an ACL injury. Thus, in this group, surgery could have modified the biomechanical patterns. Currently no studies have used as a sample injured players who have already suffered a knee injury based on the valgus collapse mechanism, causing damage to the ACL or another joint structure with the same injury mechanism, which has been treated without surgical intervention. We hypothesized that female futsal players with previous knee injury based on the valgus collapse mechanism, without surgical intervention, had kinematic patterns similar to those identified as risk patterns for ACL injury, in a change-of-direction task. Therefore, this study aimed to identify kinematic differences at initial contact between female futsal players with and without previous knee injury, in a motor pattern test including change of direction, stabilization, and acceleration. A secondary aim was to determine the kinematic differences between the DL and NDL for the whole group, in the same tasks.

## 2. Materials and Methods

### 2.1. Study Design

This study has a cross-sectional design. The allocation ratio was [1:1] between two groups. Allocation depended on clinical history: previous knee injury (KI) involving the valgus collapse mechanism without surgical intervention (injury group), or no injury (control group). The Research Ethics Committee of Community of Aragón approved this study (code PI20/127), which observed the ethical principles of the Declaration of Helsinki (64th WMA General Assembly, Fortaleza, Brazil, October 2013) [28].

### 2.2. Sample Size

The sample size was calculated based on the outcomes of previous studies [19,29]. The main variable used for sample size calculation was dynamic knee valgus at the first contact. The sample size was calculated with the GRANMO 7.12 calculator (Institut Municipal d’Investigació Mèdica, Barcelona, Spain) (https://www.imim.es/ofertadeserveis/software-public/granmo/, accessed on 1 November 2021), with an alpha risk of 0.05, a beta risk of 0.20, and the two-side test. We used a common standard deviation of 5.1 degrees [29] and a minimum expected difference of 8.4 degrees [19], estimating a follow-up loss of 20%. A total sample of 16 subjects (8 per group) was obtained.

### 2.3. Participants

The president of the Real Federación de Fútbol de Ceuta was contacted regarding participation in the study. He contacted all the national female futsal players registered in the federation, of whom 16 volunteered to participate in the study. The average age was 23.4 + 5.03 years and the average height was 1.62 ± 0.06 m. They had an active national futsal licence, and they had attended futsal training for more than 4 h per week for at least 4 months. Players were excluded if they had had an injury incompatible with regular training in the past 4 months. All the players gave written informed consent prior to their participation in the study.

### 2.4. Procedure

The first part of the evaluation was a survey to collect the individual clinical history of each participant. The data from the clinical history were used to allocate the subjects into the KI or control group. All players who had suffered a knee injury involving the valgus collapse non-contact mechanism, without surgical intervention, were included in the KI group [16,17,30]. The recorded information included age, height, weight, knee injury history, lower limb dominance, injury mechanism, medical diagnosis, pain region, time to recovery, treatment received, possible relapse, and futsal level league at the time the injury occurred. Eight players were allocated to each group.

The data collection was conducted in one session. Before the test procedure, a short mobility and warm-up activity was carried out.

The evaluation protocol included the functional change-of-direction and acceleration test (CODAT) [31,32]. This was conducted for each lower limb dominant (DL) and non-dominant (NDL). Limb dominance criteria was defined as the preferred kicking limb [25]. A 3D motion capture system (Qualisys AB, Göteborg, Sweden) was used to analyze the kinematics of change of direction with a full body model marker set without head and upper extremities. Twenty-six reflective markers were placed with adhesive tape on the players’ skin on both sides of the lower limbs and trunk. The palpation of the reflective markers was carried out following anatomical references. A force plate (Optima HPS464508-2000, AMTI, Watertown, MA, USA) was used to record the maximal torque of the ground reaction force during the initial contact phase.

The 26 markers were placed to make a static picture. The locations were at the first and fifth metatarsal head, second metatarsophalangeal, medial and lateral malleolus, large posterior surface of calcaneus, lateral and medial femoral epicondyle, anterior and posterior superior iliac spine, acromioclavicular joints, inferior scapula angle, and thoracic spinous process of T3 and T12. In addition, a cluster with four markers was placed in the lateral of the shank and thigh of both limbs. Finally, another cluster with three markers was fixed to the lateral part of the pelvic girdle on both sides. After calibration in order for the computer to recognize precisely the situation of the cameras and all the markers to create a static record for the CODAT test performance, the malleolus, epicondyles, posterior superior iliac spine, and acromioclavicular joint markers were removed from the player to avoid interference with the maximal speed task. The set-up of the markers was in line with the recommendations of Codamotion system protocols (Charnwood Dynamics Ltd., Leicestershire, UK).

**Figure 1:**
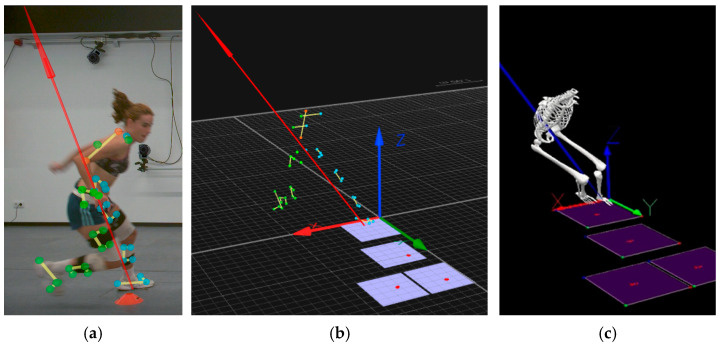
Capture moment of kinematic data during CODAT test: (**a**) Capture moment from a 2D camera that shows the player’s task; (**b**) Signal from 12 infrared high-speed cameras being processed with Visual3D software; (**c**) Final avatar processed by Virtual 3D software.

The reflective marker locations were registered through 12 infrared high-speed cameras at a rate of 250 Hz. The calibration of the space was conducted with a wand (with a length of 751.1 mm) before each data collection and the standard deviations of the wand’s length measurements were below 0.5 mm. Visual3D software (C-Motion Inc., Germantown, MD, USA) was used to analyze the change-of-direction task (Figure 1).

**Figure 2:**
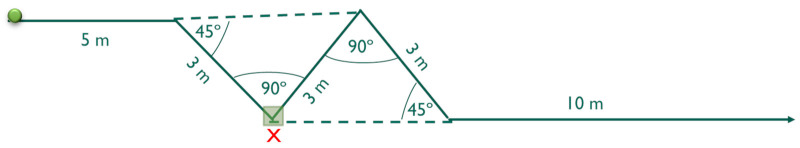
CODAT test diagram. The initial point of the test is drawn with a green ball. The green square shows the location of the force plate. The red cross identifies the change-of-direction task that was recorded with the 3D motion capture system.

Players carried out the CODAT test (Figure 2). This is a specific test that combines a sprint mechanism with the stabilization and acceleration needed for a change of direction. It involves placing a high load onto the knee in a similar way to tasks performed during training or a match [31,32,33]. Moreover, most injuries involving non-contact mechanisms occur in defensive roles, so we performed the CODAT test without a ball to recreate the defensive role during the match [13]. The CODAT test integrates a four-diagonal change-of-direction task, two of 45° and the other two of 90°, mixed with 3-m sprints and followed by a 10-m sprint. All the tests were performed at maximal speed. Prior research has observed that the average time of each sprint in soccer is 2 s and that the average distance is 10 m, which agrees with the maximal distance in the CODAT test [31]. Moreover, a 3-m sprint allows for a complete gait cycle before the change-of-direction task.

### 2.5. Outcome Variables

Since the risk of ACL injury is highest during the initial contact phase, only the moment of the maximal ground reaction torque forces at the initial contact phase in the 90° change of direction was analysed [34] (Figure 2).

Mean and standard deviation (in degrees) of the flexion/extension, adduction/abduction and internal/external rotation of the trunk, pelvis, hip, and knee were computed for the DL and NDL. For the variables in the sagittal plane projection angle (*x*-axis), positive values (>0) refer to flexion, and negative values (<0) refer to extension. In the frontal plane projection angle (*y*-axis), positive values (>0) refer to trunk inclination towards the non-supporting limb side (contralateral), contralateral pelvic tilt, hip abduction, and knee varus; and negative values (<0) refer to the trunk inclination towards the supporting limb side (ipsilateral), ipsilateral pelvic tilt, hip adduction, and knee valgus. In the transversal plane projection angle (*z*-axis), positive values (>0) refer to the non-supporting limb side, contralateral trunk rotation, contralateral pelvis rotation, and hip and knee internal rotation; and negative values (<0) refer to ipsilateral trunk displacement, ipsilateral pelvis rotation, and hip and knee external rotation.

### 2.6. Data Analysis

Data were analysed with SPSS software v.25 (SPSS Inc., Chicago, IL, USA). Normality was determined by the Kolmogorov–Smirnov test. To determine the differences in the kinematic angles between the KI and control groups, independent *t*-tests were used to compare the means. Furthermore, to determinate the differences in the kinematic angles between the DL and NDL, independent *t*-tests analysis was developed without distinguishing between players with and without injury. The level of statistical significance was set at *p* < 0.05. The effect size of the kinematic angles between the groups and limbs was determined using *Cohen’s d*. The following values were used to distinguish the levels of the effect size: 1 to 0.8 (strong effect size); 0.8 to 0.5 (moderate effect size); and 0.5 to 0.0 (less or no effect size).

## 3. Results

Eight players were allocated to each of the two groups, KI and control. The KI players had suffered a previous knee injury involving the valgus collapse mechanism, treated without surgical intervention, four of them in their DL and the rest in their NDL.

The independent *t*-test revealed that there were no significant differences between the groups in any kinematic parameters. However, strong *Cohen’s d* effect sizes, above 0.8, between the groups were found in the hip adduction, hip rotation, and pelvis rotation in the dominant limb (Table 1). The KI group had higher hip adduction than the control group (14.15 ± 4.64 degrees versus 10.69 ± 4.92 degrees in the control group) (*Cohen’s d* = 0.82). The KI group presented a hip internal rotation of 1.26 ± 14.68 degrees, versus 11.55 ± 14.29 degrees of hip external rotation in the control group (*Cohen’s d* = 0.88). The KI group showed a contralateral pelvis rotation of 6.11 ± 8.21 degrees, versus 1.35 ± 5.67 degrees of ipsilateral pelvis rotation in the control group (*Cohen’s d* = 1.06) (Table 1).

The analysis based on limb dominance taking both groups together was significant only for the knee valgus variable. Knee valgus was higher in the DL (9.02 ± 7.31 degrees versus 1.27 ± 9.05 degrees in the NDL) (*p* < 0.05), with a strong effect size (*Cohen’s d* = 0.94) (Table 2). All the other variables showed similar values between the DL and NDL.

## 4. Discussion

The main purpose of this study was to investigate kinematic differences at initial contact between female futsal players with and without previous knee injury, in a motor-pattern test including change of direction, stabilization, and acceleration. None of the variables showed significant differences between female futsal players with and without previous knee injury. However, a strong effect size between groups was obtained in some kinematics in the DL. With respect to the secondary aim of this study, to determine kinematic differences between the DL and NDL for the whole group, only knee valgus was significantly higher in the DL.

Knee valgus was significantly higher in the DL (9.02 ± 7.31 degrees) than in the NDL (1.27 ± 9.05 degrees) (*p* = 0.049) in the whole group. As other studies have explained, the ACL usually breaks at an average of 13 degrees of knee valgus at initial contact and 22 degrees of knee valgus at the moment of injury, 48 milliseconds after the initial contact [6]. In our findings, the KI and control players were near to these values at initial contact in the DL, without significant differences between them (KI: 10.58 ± 5.64 degrees; control group: 8.87 ± 8.45). However, in the NDL, the values were much lower (KI: 0.15 ± 10.99 degrees; control group: 2.87 ± 8.01). Knee valgus is one of most relevant risk factors in ACL non-contact injury mechanisms, and 88% to 100% of the knee injuries in female soccer are induced by non-contact mechanisms [6,7]. As observed in the pivot shift test, during a valgus knee movement, the femur moves medially relative to the tibia, which causes the lateral condyle to slide into the medial side. These movements cause medial yawing of the knee that increases the ACL load. If the load on the ACL is too high, it might break and cause subluxation of the tibia, usually at around 30 degrees of knee flexion that reduces in deeper flexion [24,35]. Recently, Di Paolo et al. [36] and Collings et al. [37] have verified that higher knee valgus values in different tasks were associated with future noncontact ACL injury in elite female soccer players. Our results showed a higher ACL injury risk position in the DL than in the NDL. DeLang et al. [25], in their meta-analysis, explain that soccer players are more likely to suffer injuries in their DL regardless of their playing level or gender. This is due to two main reasons. Firstly, there is neuromuscular fatigue in the DL as a result of the maximal effort involved in the kicking activity and the high-intensity stabilization actions. In futsal, the DL also accumulates more burden than the NDL. Futsal is a sport with changes of intensity and direction each 3.3 s on average. Therefore, 26% of the match is played at high intensity [26]. This fact produces fatigue in both limbs, but the dominant one is also the kicking leg. Studies explain that players receive the ball twice a minute on average, and 84% of this is with the DL [25,38]. In this way, the DL accumulates fatigue relative to the intensity of the game with additional fatigue due to the maximal effort of kicking. Secondly, there are intrinsic (capability, comfortability, or awareness) and extrinsic factors (opponent position or game situation) that make it necessary to use the DL as the stabilization limb, and it may not be used to these actions [25]. Furthermore, most ACL injuries occur in defensive “pressing/tackling” or offensive duel “tackling” situations. These are high-speed actions and are driven by the match conditions, in which player does not have enough time to decide on the use of the DL or NDL. These forced actions are the most likely to result in injury. In contrast, both groups presented better knee valgus values in the NDL; this might be due to better neuromuscular control in this limb, since it is more used to the stabilization task.

“Valgus collapse” has been defined as the combination of hip adduction, hip internal rotation, and knee abduction. In the current study, in the DL, the limb which had a level of knee valgus near to the injury risk threshold, the KI players had more hip adduction than the control players in the change-of-direction task (*Cohen’s d*: 0.82). This was explained by the fact that when the KI players carried out a task that promoted high knee valgus values, their stabilization pattern was pathological, as explained by what is described in the literature as the “perfect storm” of ACL injury mechanism [15,16,19]. These results agree with previous research, such as the study by Dix et al. [39], who verified that players who suffered ACL injury had a higher “valgus collapse”, a combination of knee valgus, hip adduction, and internal hip rotation, during preseason testing, before ACL injury. The authors noted significant differences between injured and non-injured players only in the hip adduction variable. This study developed prospective data collection in national female soccer players, comparing initial kinematic values in a change-of-direction task of 90° between groups. Therefore, the hypothesis of our KI group performing a kinematic pattern similar to the ACL injury pattern is confirmed.

Hip and knee internal rotation are involved in knee valgus definition, as explained in video analysis research [6,14,40]. In the current study, both groups had knee internal rotation at the evaluation moment, but the KI players had hip internal rotation in contrast to control players who developed hip external rotation (*Cohen’s d*: 0.88). Hip internal rotation, combined with hip adduction and knee abduction, increases structural stress on the ACL due to its anatomical situation from the front internal tibial surface to the back external femur condyle [11,39,41]. Therefore, if the hip internal rotation range of movement is increased, the strain fibres are at maximum stress and are broken easily. At the same time, increases in knee abduction and hip adduction cause medial displacement of the center of mass [24,39,41], and the joining of both conditions facilitates ACL risk injury. As is explained by Lucarno et al. [6] in their systematic video analysis, 80% of the non-contact ACL injuries that occurred during matches in female players in six of the top leagues in the Federation International of Football Association (FIFA) Women’s World Ranking were associated with hip internal rotation movement. Koga et al. show the same result in female players of other sports, such as basketball or handball. Our results showed that the KI players had a greater hip internal rotation range of motion than the control players, in agreement with previous research. Furthermore, hip internal rotation might be the result of weakness in the hip external rotation muscles., especially the gluteus maximus, which becomes weak due to accumulated fatigue. Fatigue could be the result of carrying out many functions at the same time. As strong external rotation is an important factor in avoiding knee valgus, we can say that it is a protective factor of ACL injury involving non-contact mechanisms. In fact, our control players showed hip external rotation in the DL during the change-of-direction task.

All the players in our study, in both the KI and control groups, had a contralateral trunk rotation from the supporting limb. Many authors have demonstrated that if the contralateral trunk rotation increases, the knee valgus increases too, raising the risk of ACL injury [6,14,16,42]. As Della Villa et al. [14] shows in their research, 53% of the players with ACL injuries had contralateral trunk rotation at the moment of injury, especially during pressing and landing tasks. As Lloyd et al. [16] explain in their “perfect storm” definition of ACL injury mechanisms, contralateral rotation causes a medial displacement of the center of mass, which increases the loading on the medial knee compartment. However, no prior authors have studied the influence of pelvic rotation. In our study, the KI players performed a contralateral pelvic rotation following the trunk motion direction. On the contrary, members the control group in whom contralateral trunk rotation existed realized an ipsilateral pelvic rotation to the DL (*Cohen’s d*: 1.06). This might be a balance mechanism to counteract the opposite trunk rotation, and to decelerate the medial velocities suffered by the knee as a result of the trunk rotation movement [43]. It would be interesting to develop studies to explore this hypothesis in the future.

### Limitations

This study is limited by the relatively small sample size and by the cross-sectional design conducted in a single geographic location; thus, any causality can be referred to the relationship in the relevant results. Due to the study design, it was not possible to state whether the worst kinematic patterns observed in the KI group were present before the knee injury, making the injury more likely to occur, or whether they existed as a result of the disfunctions induced by the injury. A future larger cohort and prospective studies might support our findings and develop new analysis in pelvic kinematics. However, the current findings may be relevant for the development of specific preventive training in female soccer players. The preventive training should be based on intensive neuromuscular training to avoid fatigue of the muscles, especially of the DL, and in the reinforcement of the hip external rotation muscles.

## 5. Conclusions

The current study found kinematic differences at initial contact between female futsal players with and without previous knee injury, in a motor pattern test. The players with no previous history of knee injury had a more physiological position to avoid the valgus collapse mechanism in hip adduction and internal rotation, and in the pelvis rotation, in the dominant limb. The players with previous injury had a kinematic pattern closer to those related to LCA injury, which might have existed before the injury, making it more likely to occur, or as a result of the disfunctions induced by the injury. All the players showed more knee valgus in their dominant limb, which is the limb with a greater risk of injury, revealing the clinical relevance of neuromuscular stabilization training for this limb.

## Figures and Tables

**Table 1:** Comparative analysis of the kinematic outcomes between KI and control groups in DL and NDL.

	Dominant Limb					Non-Dominant Limb				
	KI	Control				KI	Control			
	Mean ± SD	Mean ± SD	*p*-Value	*Cohen’s d*	CI (95%)	Mean ± SD	Mean ± SD	*p*-Value	*Cohen’s d*	CI (95%)
Knee Flexion (°) (+flexion/−extension)	29.64 ± 9.74	29.60 ± 9.87	0.994	0.00	(−10.93–11.01)	26.54 ± 20.44	35.50 ± 12.21	0.314	0.53	(−27.44–9.52)
Pelvis Flexion (°) (+flexion/−extension)	10.30 ± 10.24	13.38 ± 12.71	0.618	0.27	(−16.09–9.93)	12.93 ± 17.19	13.44 ± 10.69	0.945	0.04	(−16.10–15.22)
Trunk Flexion (°) (+flexion/−extension)	11.66 ± 4.70	12.22 ± 13.42	0.934	0.06	(−15.07–13.93)	14.04 ± 18.84	11.41 ± 12.87	0.754	0.16	(−15.15–20.41)
Knee Valgus (°) (+varus/−valgus)	−10.58 ± 5.64	−8.87 ± 8.45	0.657	0.24	(−15.79–(−5.36))	−0.15 ± 10.99	−2.87 ± 8.01	0.59	0.28	(−9.57–3.83)
Hip Adduction (°) (+abduction/−adduction)	−14.15 ± 4.64	−10.69 ± 4.92	0.184	0.82 **	(−8.84–1.87)	−14.02 ± 15.00	−15.82 ± 13.36	0.810	0.12	(−14.02–17.60)
Pelvis tilt (°) (+contralateral/−ipsilateral)	−6.24 ± 9.31	−10.57 ± 8.02	0.350	0.50	(−14.85–2.36)	−7.67 ± 8.82	−2.28 ± 15.77	0.439	0.42	(−15.47–10.90)
Trunk displacement (°) (+contralateral/−ipsilateral)	−12.23 ± 8.97	−12.05 ± 14.30	0.977	0.01	(−20.53–(−3.94))	−14.18 ± 9.66	−8.27 ± 10.98	0.292	0.57	(−17.44–0.91)
Knee rotation (°) (+internal/−external)	6.36 ± 10.74	1.85 ± 9.30	0.399	0.45	(−3.57–16.29)	5.53 ± 8.20	4.99 ± 4.82	0.877	0.08	(−4.25–6.21)
Hip rotation (°) (+internal/−external)	1.26 ± 14.68	−11.55 ± 14.29	0.111	0.88 **	(−12.31–14.84)	−8.97 ± 11.74	−1.17 ± 12.65	0.314	0.64	(−11.75–9.40)
Pelvis rotation (°) (+contralateral/−ipsilateral)	6.11 ± 8.21	−1.35 ± 5.67	0.059	1.06 **	(−13.70–1.48)	1.82 ± 14.01	8.52 ± 11.71	0.331	0.52	(−18.31–1.27)
Trunk rotation (°) (+contralateral/−ipsilateral)	3.22 ± 9.29	7.81 ± 12.90	0.450	0.41	(−11.81–5.38)	1.62 ± 9.96	5.89 ± 10.12	0.426	0.42	(−14.35–2.57)

** Effect size strong to excellent *d* > 0.8.

**Table 2:** Comparative analysis of the kinematic outcomes between the DL and the NDL in the whole group.

	DL	NDL			
	Mean ± SD	Mean ± SD	*p*-Value	*Cohen’s d*	CI (95%)
Knee Flexion (°) (+flexion/−extension)	31.12 ± 10.93	32.62 ± 16.84	0.757	0.11	(−9.33–6.48)
Hip Flexion (°) (+flexion/−extension)	46.85 ± 37.07	41.98 ± 21.01	0.559	0.16	(−8.92–9.74)
Pelvis Flexion (°) (+flexion/−extension)	13.92 ± 13.50	15.35 ± 15.66	0.706	0.10	(−8.41–14.96)
Trunk Flexion (°) (+flexion/−extension)	10.66 ± 13.16	10.25 ± 17.67	0.926	0.03	(−11.61–8.62)
Knee Valgus (°) (+varus/−valgus)	−9.02 ± 7.31	−1.27 ± 9.05	0.049 *	0.94 **	(−15.46–(−0.04))
Hip Adduction (°) (+abduction/−adduction)	−13.04 ± 5.64	−14.85 ± 13.20	0.624	0.18	(−5.90–9.52)
Pelvis tilt (°) (+contralateral/−ipsilateral)	−9.23 ± 8.76	−5.62 ± 12.85	0.449	0.33	(−14.85–2.36)
Trunk displacement (°) (+contralateral/−ipsilateral)	−12.47 ± 11.37	−11.35 ± 10.20	0.826	0.10	(−11.66–9.70)
Knee rotation (°) (+internal/−external)	4.36 ± 9.70	5.11 ± 6.17	0.809	0.09	(−3.57–16.29)
Hip rotation (°) (+internal/−external)	−5.92 ± 14.98	−4.94 ± 12.05	0.847	0.07	(−11.66–9.70)
Pelvis rotation (°) (+contralateral/−ipsilateral)	2.08 ± 7.47	5.72 ± 12.47	0.391	0.35	(−5.15–12.43)
Trunk rotation (°) (+contralateral/−ipsilateral)	4.96 ± 11.20	2.99 ± 10.25	0.606	0.18	(−9.92–5.99)

* Statistical significance *p* < 0.05/** Effect size strong to excellent *d* > 0.8.

## Data Availability

The datasets presented in this study are available on request from the corresponding author. All data covered by this study are included in this manuscript.
